# Supplementary material for: Combined inhibition of KRASG12C and mTORC1 kinase is synergistic in non-small cell lung cancer
Source: Nat Commun. 2024 Jul 19;15:6076. doi: 10.1038/s41467-024-50063-z (PMC11258147; doi:10.1038/s41467-024-50063-z)
Supplement: Supplementary file 1 — Supplementary Information [file 41467_2024_50063_MOESM1_ESM.pdf]

Supplementary Information

Combined inhibition of KRAS^{G12C} and mTORC1 kinase is synergistic in non-small cell lung cancer

Hidenori Kitai, Philip H. Choi, Yu C. Yang, Jacob A. Boyer, Adele Whaley, Priya Pancholi, Claire Thant, Jason Reiter, Kevin Chen, Vladimir Markov, Hirokazu Taniguchi, Rui Yamaguchi, Hiromichi Ebi, James Evans, Jingjing Jiang, Bianca Lee, David Wildes, Elisa de Stanchina, Jacqueline A. M. Smith, Mallika Singh, Neal Rosen

Contents

Supplementary Figures 1-6

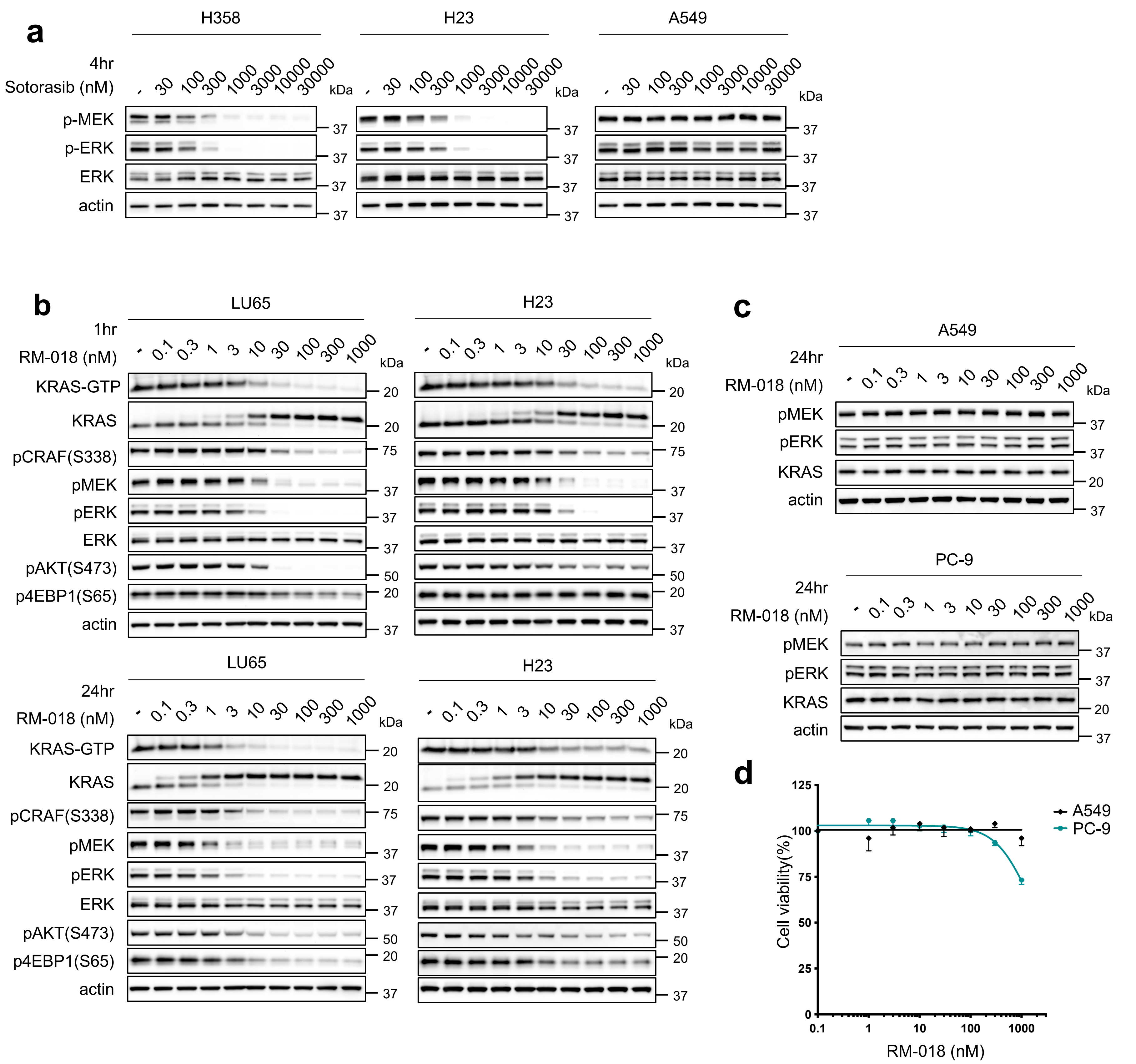

Supplementary Fig. 1. a, H358, H23, A549 cells were treated with varying doses of Sotorasib for 4 hours and lysates were analyzed by immunoblot. **b**, Lu65 and H23 cells were treated with varying doses of RM-018 for 1 hour and 24 hours and lysates were analyzed by immunoblot. **c,** A549 and PC-9 cells were treated with varying doses of RM-018 for 24 hours and lysates were analyzed by immunoblot. **d,** A549 and PC-9 cells were treated with varying doses of RM-018 for 72 hours and cell viability was measured. Data are the mean ± SD of n=8 experimental replicates.

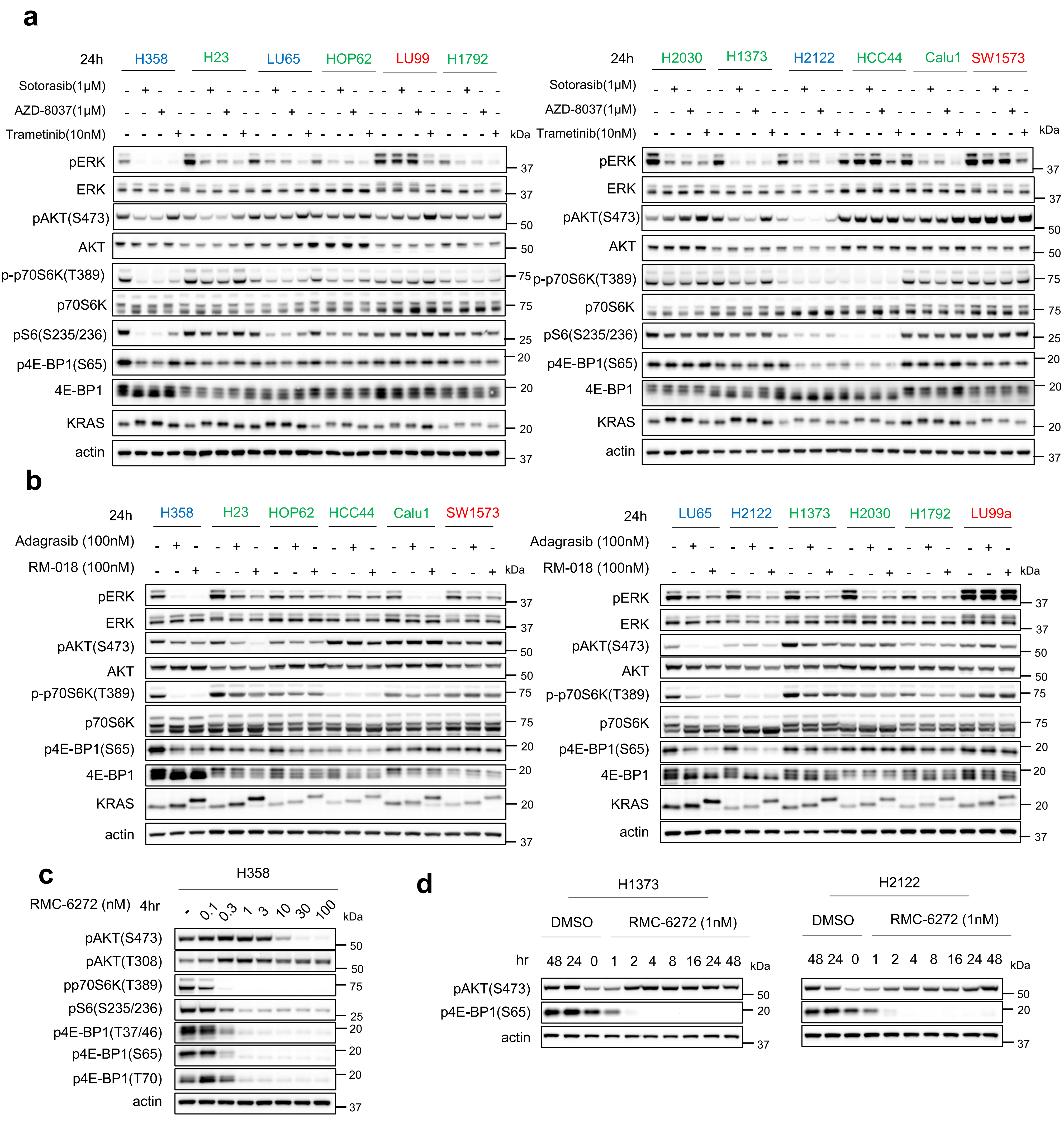

Supplementary Fig. 2. **a,** KRAS^{G12C} mutant cancer cell lines were treated with either 1 μM Sotorasib, 1 μM AZD8037, or 10 nM Trametinib for 24 hours, and lysates were analyzed by immunoblot. Cell lines are color-coded as in Fig. 2a (blue=sensitive, green=intermediate, red=resistant). **b**, KRAS^{G12C} mutant cancer cell lines were treated with either 100 nM Adagrasib or 100 nM RM-018 for 24 hours, and lysates were analyzed by immunoblot. Cell lines are color-coded as in Fig. 2a (blue=sensitive, green=intermediate, red=resistant). **c**, H358 cells were treated with varying doses of RMC-6272 for 4 hours and lysates were analyzed by immunoblot, **d,** H1373 and H2122 cell lines were treated with 1 nM RMC-6272 for the indicated times, and lysates were analyzed by immunoblot.

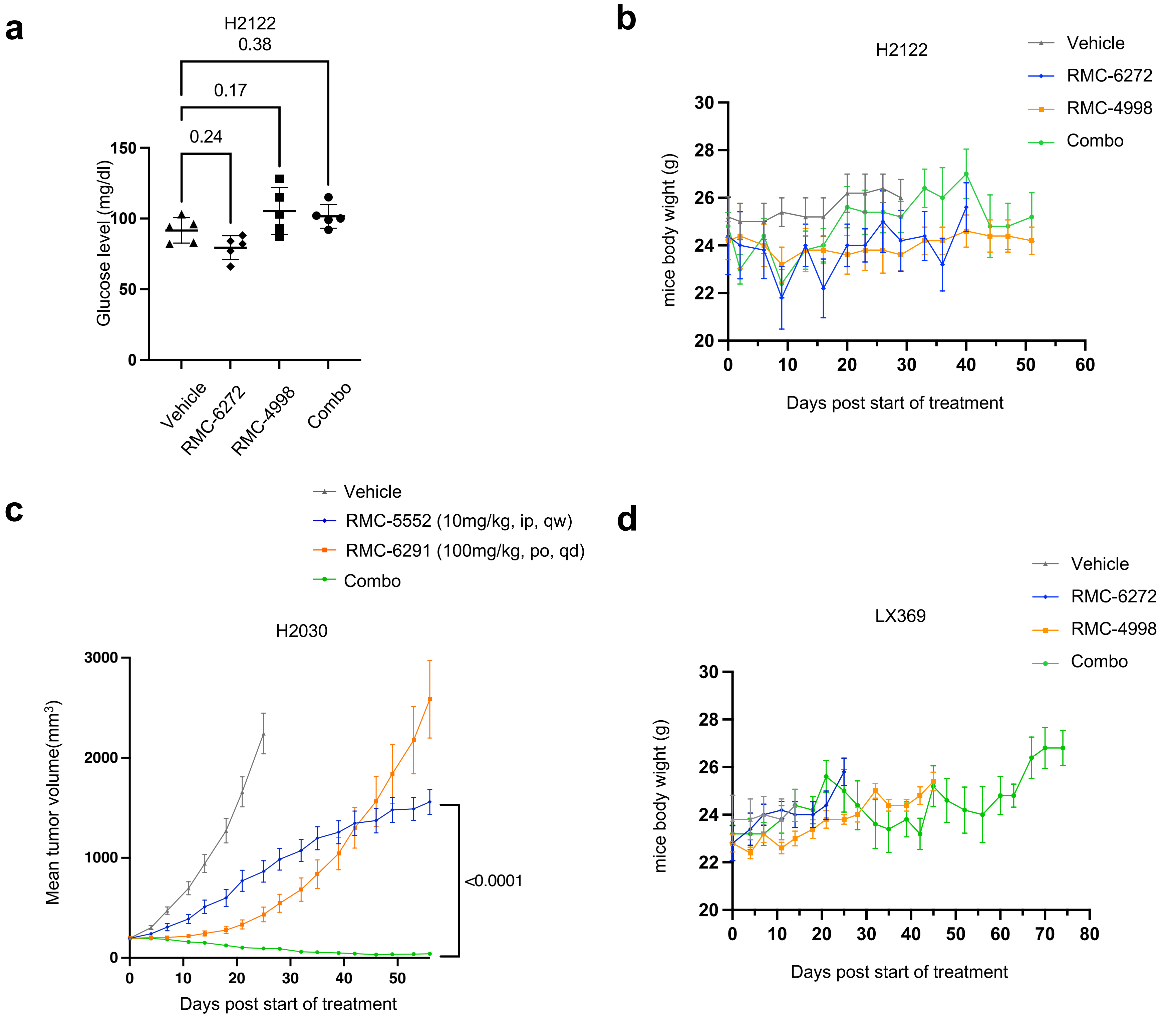

Supplementary Fig. 3. a, During the treatment period in Fig. 3A, tail snip blood samples in mice were collected once weekly followed by glucose assessment with a glucometer. The data shown represent the means ± SD of n=5 mice. P values were calculated by one-way ANOVA and post-hoc Tukey's test. **b,** Average body weights of H2122 xenograft mice treated with Vehicle, RMC-6272 6 mg/kg weekly, RMC-4998 80 mg/kg daily, or drug combination are shown relative to starting body weight. The data shown represent the means ± SEM of n=5 mice. **c,** H2030 xenografts were treated 24 days post-implant with Vehicle, RMC-6291 100 mg/kg daily by oral gavage, RMC-5552 10 mg/kg by intraperitoneal injection once weekly, or the combination. The data shown represent the mean tumor volume ± SEM of n=8 mice. P value is shown and was calculated by two-sided t-test. **d,** Average body weights of LX369 PDX bearing mice treated with Vehicle, RMC-6272 6 mg/kg weekly, RMC-4998 80 mg/kg daily, or the combination of these drugs are shown relative to starting body weight. The data shown represent the mean ± SEM of n=5 mice.

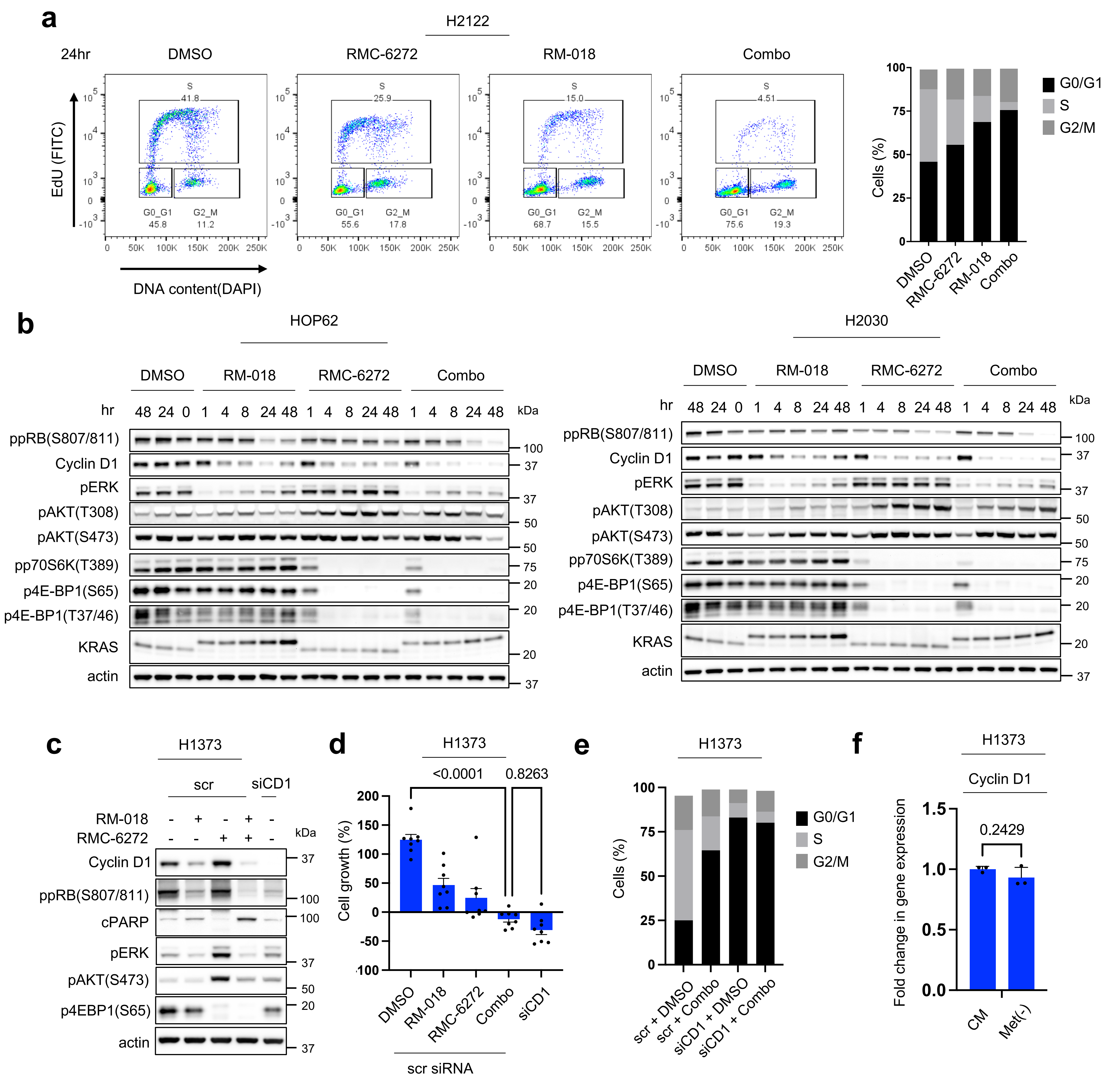

Supplementary Fig. 4. a, H2122 cells were treated with 1 nM RMC-6272, 100 nM RM-018, or the combination for 24 hours. Cell cycle states were detected with EdU-DAPI based flow cytometry. **b,** HOP62 and H2030 cell lines were treated with either 100 nM RM-018, 1 nM RMC-6272, or the combination for the indicated times, and lysates were analyzed by immunoblot. **c,** H1373 cells were transfected with siRNAs targeting Cyclin D1 (siCD1) or scramble (scr) siRNA and cultured for 24 hours. Media was then replaced with 100 nM RM-018 and/or 1 nM RM-6272 as indicated and cells were treated for an additional 48 hours. Lysates were then analyzed by immunoblot. **d,** H1373 cells were transfected with siRNAs targeting Cyclin D1 (siCD1) or scramble (scr) siRNA and cultured for 24 hours. The cells were then treated with 100 nM RM-018, 1 nM RM-6272, or the combination for 72 hours and cell growth was measured by the Cell-titer Glo assay. Data are the mean ± SD of n=8 biological replicates. P values were calculated by one-way ANOVA and post-hoc Tukey's test. **e,** H1373 cells were transfected with siRNAs targeting Cyclin D1 (siCD1) or scramble (scr) siRNA and then treated with either DMSO or the combination of 1 nM RMC-6272 and 100 nM RM-018 (Combo) for 24 hours. Cell cycle states were then detected with EdU-DAPI based flow cytometry. **f,** Cyclin D1 mRNA levels were determined by quantitative PCR in H1373 cells following 24 hours of methionine starvation. The data shown represent the means ± SD of n=3 biological replicates. P values were calculated by two-sided t-test.

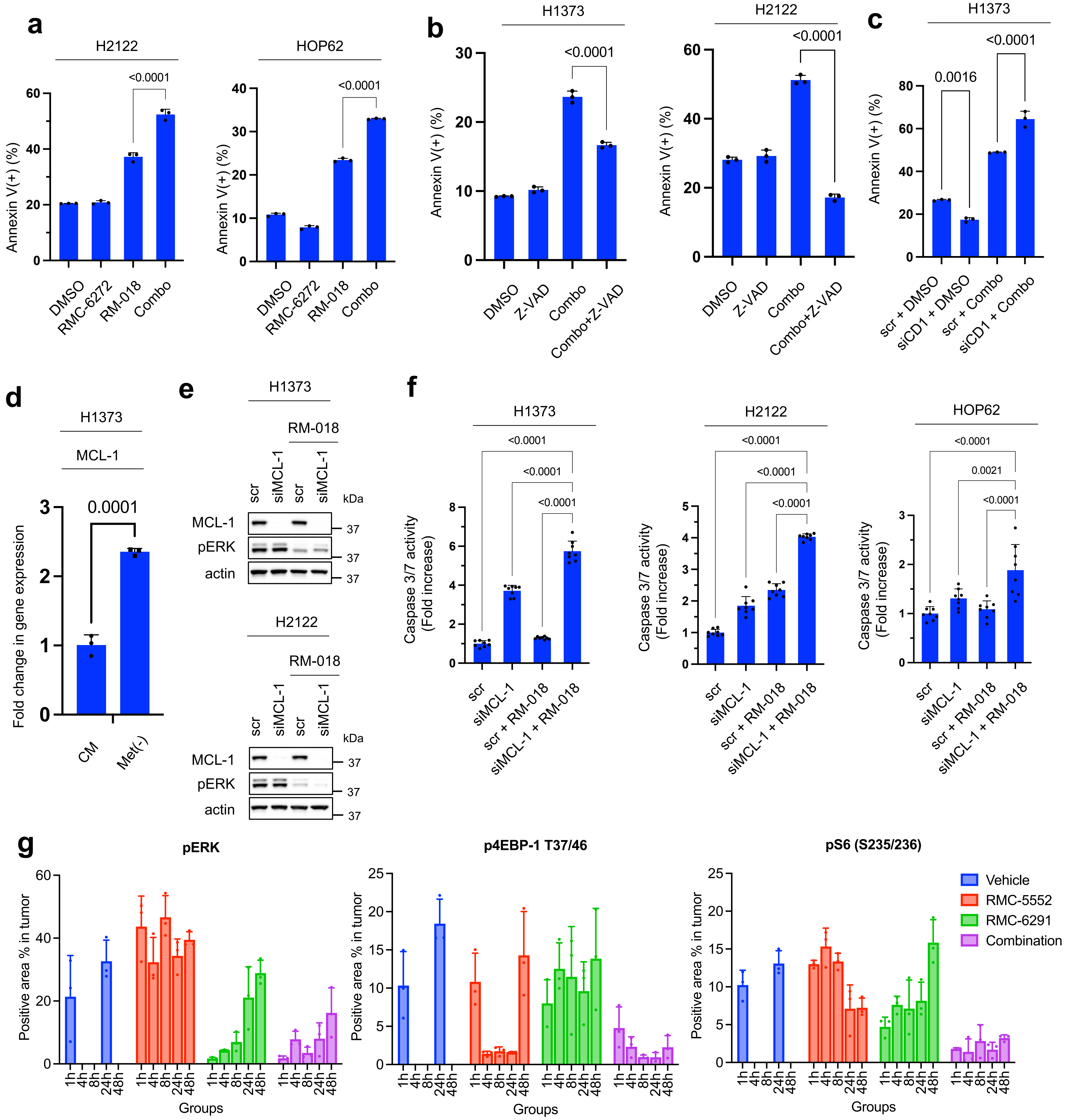

Supplementary Fig. 5. a, H2122 and HOP62 cells were treated with 100 nM RM-018, 1 nM RMC-6272, or the combination for 72 hours, and analyzed by FACS to quantify annexin V positive cells. Data are means ± SD for n=3 experimental replicates. P values were calculated by one-way ANOVA and post-hoc Tukey's test. **b,** H1373 and H2122 cell lines were treated with 100 μM Z-VAD-FMK, the combination of 1nM RMC-6272 with 100nM RM-018, and the combination with 100 μM Z-VAD-FMK for 72 hours and analyzed by FACS to quantify annexin positive cells. Data are means ± SD for n=3 experimental replicates. P values were calculated by Student's t-test. **c,** H1373 cells were transfected with siRNAs targeting Cyclin D1 (siCD1) or scramble (scr) siRNA and cultured for 24 hours. The cells were then treated with DMSO or the combination of 100 nM RM-018 and 1 nM RMC-6272 (Combo) for 72 hours and analyzed by FACS to quantify annexin V positive cells. Data are means ± SD for n=3 experimental replicates. P values were calculated by one-way ANOVA and post-hoc Tukey's test. **d,** MCL-1 mRNA levels were determined by quantitative PCR in H1373 cells following 24 hours of methionine starvation. The data shown represent the means ± SD of n=3 experimental replicates. P values were calculated by Student's t-test. **e,** H1373 and H2122 cells were transfected with siRNAs targeting MCL-1 or scramble (scr) siRNA and cultured for 24 hours. Media was then replaced with or without 100 nM RM-018 and cells were treated for an additional 24 hours. Lysates were then analyzed by immunoblot. **f,** Caspase 3/7 activity after siRNA knockdown of MCL-1 or siRNA control (scr) for 24 hours, followed by treatment with 100 nM RM-018 for 24 hours in H1373, H2122 and HOP62 cells. Data are means ± SD for n=8 experimental replicates. P values were calculated by one-way ANOVA and post-hoc Tukey's test. **g,** Quantification of IHC analysis of indicated proteins as shown in Fig 5G. The data shown represent the means ± SD of n=3 experimental replicates.

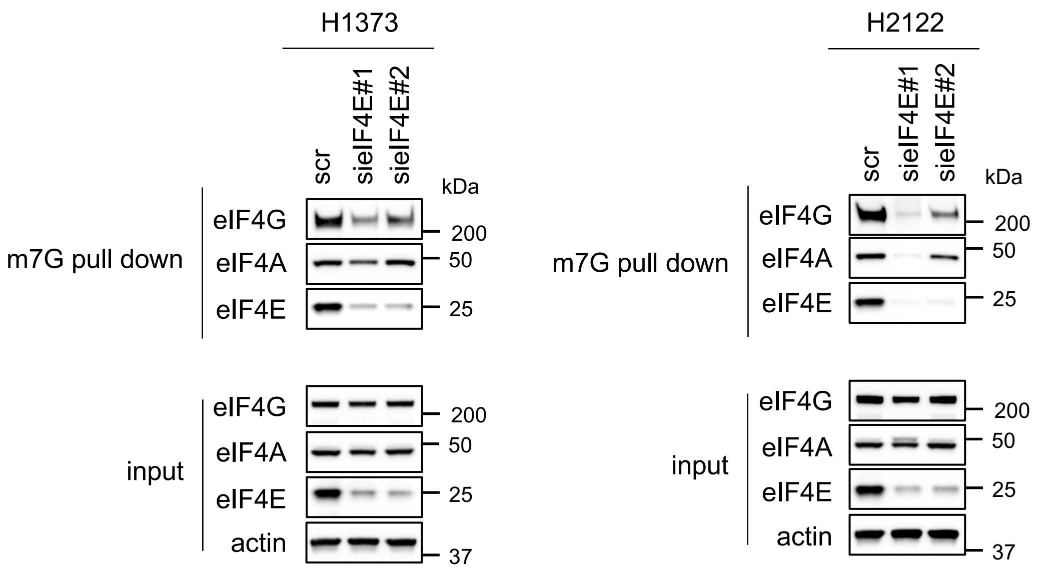

Supplementary Fig. 6. H1373 and H2122 cells were transfected with two different siRNAs targeting eIF4E or scramble (scr) siRNA and cultured for 72 hours. The cell lysates were then incubated with m^7GTP-conjugated beads, and then analyzed by immunoblot in parallel with whole cell extracts (input).
