# Supplementary material for: Combined inhibition of KRASG12C and mTORC1 kinase is synergistic in non-small cell lung cancer
Source: Nat Commun. 2024 Jul 19;15:6076. doi: 10.1038/s41467-024-50063-z (PMC11258147; doi:10.1038/s41467-024-50063-z)
Supplement: Supplementary file 2 — Reporting Summary [file 41467_2024_50063_MOESM2_ESM.pdf]

nature portfolio

Corresponding author(s): Neal Rosen

Last updated by author(s): Jun 5, 2024

Reporting Summary

Nature Portfolio wishes to improve the reproducibility of the work that we publish. This form provides structure for consistency and transparency in reporting. For further information on Nature Portfolio policies, see our Editorial Policies and the Editorial Policy Checklist.

Statistics

For all statistical analyses, confirm that the following items are present in the figure legend, table legend, main text, or Methods section.

n/a	Confirmed	
☐	☒	The exact sample size (*n*) for each experimental group/condition, given as a discrete number and unit of measurement
☐	☒	A statement on whether measurements were taken from distinct samples or whether the same sample was measured repeatedly
☐	☒	The statistical test(s) used AND whether they are one- or two-sided<br>*Only common tests should be described solely by name; describe more complex techniques in the Methods section.*
☐	☒	A description of all covariates tested
☐	☒	A description of any assumptions or corrections, such as tests of normality and adjustment for multiple comparisons
☐	☒	A full description of the statistical parameters including central tendency (e.g. means) or other basic estimates (e.g. regression coefficient) AND variation (e.g. standard deviation) or associated estimates of uncertainty (e.g. confidence intervals)
☐	☒	For null hypothesis testing, the test statistic (e.g. *F*, *t*, *r*) with confidence intervals, effect sizes, degrees of freedom and *P* value noted<br>*Give P values as exact values whenever suitable.*
☒	☐	For Bayesian analysis, information on the choice of priors and Markov chain Monte Carlo settings
☒	☐	For hierarchical and complex designs, identification of the appropriate level for tests and full reporting of outcomes
☐	☒	Estimates of effect sizes (e.g. Cohen's *d*, Pearson's *r*), indicating how they were calculated

Our web collection on statistics for biologists contains articles on many of the points above.

Software and code

Policy information about availability of computer code

Data collection	No custom algorithm or software was generated in this study
Data analysis	No custom algorithm or software was generated in this study

For manuscripts utilizing custom algorithms or software that are central to the research but not yet described in published literature, software must be made available to editors and reviewers. We strongly encourage code deposition in a community repository (e.g. GitHub). See the Nature Portfolio guidelines for submitting code & software for further information.

Data

Policy information about availability of data

All manuscripts must include a data availability statement. This statement should provide the following information, where applicable:

- Accession codes, unique identifiers, or web links for publicly available datasets
- A description of any restrictions on data availability
- For clinical datasets or third party data, please ensure that the statement adheres to our policy

All data included in this study is provided in the article, supplementary information, or source data file.

Research involving human participants, their data, or biological material

Policy information about studies with human participants or human data. See also policy information about sex, gender (identity/presentation), and sexual orientation and race, ethnicity and racism.

Reporting on sex and gender	N/A
Reporting on race, ethnicity, or other socially relevant groupings	N/A
Population characteristics	N/A
Recruitment	N/A
Ethics oversight	N/A

Note that full information on the approval of the study protocol must also be provided in the manuscript.

Field-specific reporting

Please select the one below that is the best fit for your research. If you are not sure, read the appropriate sections before making your selection.

☒ Life sciences ☐ Behavioural & social sciences ☐ Ecological, evolutionary & environmental sciences

For a reference copy of the document with all sections, see nature.com/documents/nr-reporting-summary-flat.pdf

Life sciences study design

All studies must disclose on these points even when the disclosure is negative.

Sample size	Samples size was determine based on the expected effect size and the statistical power needed to determine significance
Data exclusions	No data exclusions
Replication	When described as such in the manuscript, experiments were done multiple times and a representative example is shown
Randomization	Mice were randomly distributed into treatment groups
Blinding	No blinding was done

Reporting for specific materials, systems and methods

We require information from authors about some types of materials, experimental systems and methods used in many studies. Here, indicate whether each material, system or method listed is relevant to your study. If you are not sure if a list item applies to your research, read the appropriate section before selecting a response.

Materials & experimental systems

n/a	Involved in the study	
☐	☒	Antibodies
☐	☒	Eukaryotic cell lines
☒	☐	Palaeontology and archaeology
☐	☒	Animals and other organisms
☒	☐	Clinical data
☒	☐	Dual use research of concern
☒	☐	Plants

Methods

n/a	Involved in the study	
☒	☐	ChIP-seq
☐	☒	Flow cytometry
☒	☐	MRI-based neuroimaging

Antibodies

Antibodies used	Primary antibodies obtained from Cell Signaling Technologies and used at 1:1000 dilution: pERK (#4370), ERK (#4696), pAKT T308 (#2965), pAKT S473 (#4060), AKT (#9272), p4EBP1 S65 (#9451), p4EBP1 T37/46 (#9459), 4EBP1 (#9452), p-p70S6K (#9234), p70S6K (#34475), pCRAF C338 (#9427), pMEK (#9154), CyclinD1 (#55506), ppRB (#8516), MCL-1 (#5453), BIM (#2933), PUMA (#12450), cleaved PARP (#5625), cleaved Caspase-3 (#9661), eIF4G (#2498), eIF4A (#2013), eIF4E (#2067), pEIF4E (#9741), Actin (#4970), BCL-XL (#2764). KRAS primary antibody was obtained from LSBio (#LS-C1765665).

Validation	Validation as per manufacturer

Eukaryotic cell lines

Policy information about cell lines and Sex and Gender in Research

Cell line source(s)	The lung cancer cell lines NCI-H358, NCI-H2122, NCI-H2030, HCI-H1373, NCI-H1792, NCI-H23, HOP62, Calu-1, and SW1573, A549 were purchased from the American Type Culture Collection. PC-9 was provided from Emily Cheng Lab (MSKCC). HCC44 was obtained from Korean cell line bank. LU-65 and LU-99A were obtained from the Japanese Cell Research Bank (Osaka, Japan).
Authentication	No validation was performed
Mycoplasma contamination	All cell lines tested negative for Mycoplasma
Commonly misidentified lines (See ICLAC register)	N/A

Animals and other research organisms

Policy information about studies involving animals; ARRIVE guidelines recommended for reporting animal research, and Sex and Gender in Research

Laboratory animals	For the CDX experiments, athymic strain #007850 6-10 week old female mice were used. For the PDX experiments, NSG strain #005557 6-10 week old female mice were used.
Wild animals	N/A
Reporting on sex	Only female mice were used.
Field-collected samples	N/A
Ethics oversight	MSK Animal Use and Care Committee

Note that full information on the approval of the study protocol must also be provided in the manuscript.

Plants

Seed stocks	N/A
Novel plant genotypes	N/A
Authentication	N/A

Flow Cytometry

Plots

Confirm that:

- [x] The axis labels state the marker and fluorochrome used (e.g. CD4-FITC).
- [x] The axis scales are clearly visible. Include numbers along axes only for bottom left plot of group (a 'group' is an analysis of identical markers).
- [x] All plots are contour plots with outliers or pseudocolor plots.
- [x] A numerical value for number of cells or percentage (with statistics) is provided.

Methodology

Sample preparation	5-10×105 cells were plated in 10 cm dishes and treated with DMSO, 1nM RMC-6272, 100 nM RM-018 or combination for 24 hours. Before harvesting, cells were incubated with 10 µM EdU for two hours. The cells were harvested stained with Click-iT EdU Alexa Fluor 488 Flow Cytometry Assay Kit (Invitrogen) according to the manufacturer's protocol.
Instrument	Aurora Cytek

Software	FlowJo
Cell population abundance	10,000 cells per sample. At least 500 cells per gating fraction.
Gating strategy	Preliminary SSC-A and FSC-A gates, followed by SSC-H and SSC-W, and then FSC-H and FSC-W. Gating boundaries were determined by negative and positive controls.

☐ Tick this box to confirm that a figure exemplifying the gating strategy is provided in the Supplementary Information.
